# Mitochondrial Aging and Age-Related Dysfunction of Mitochondria

**DOI:** 10.1155/2014/238463

**Published:** 2014-04-10

**Authors:** Dimitry A. Chistiakov, Igor A. Sobenin, Victor V. Revin, Alexander N. Orekhov, Yuri V. Bobryshev

**Affiliations:** ^1^Department of Medical Nanobiotechnology, Pirogov Russian State Medical University, Moscow 117997, Russia; ^2^Laboratory of Medical Genetics, Russian Cardiology Research and Production Complex, Moscow 121552, Russia; ^3^Laboratory of Cellular Mechanisms of Atherogenesis, Institute of General Pathology and Pathophysiology, Russian Academy of Medical Sciences, Moscow 125315, Russia; ^4^Biological Faculty, N.P. Ogaryov Mordovian State University, Saransk 430005, Russia; ^5^Institute for Atherosclerosis Research, Skolkovo Innovative Center, Moscow 143025, Russia; ^6^Faculty of Medicine, University of New South Wales, Sydney, NSW 2052, Australia; ^7^School of Medicine, University of Western Sydney, Campbelltown, NSW 2560, Australia

## Abstract

Age-related changes in mitochondria are associated with decline in mitochondrial function. With advanced age, mitochondrial DNA volume, integrity and functionality decrease due to accumulation of mutations and oxidative damage induced by reactive oxygen species (ROS). In aged subjects, mitochondria are characterized by impaired function such as lowered oxidative capacity, reduced oxidative phosphorylation, decreased ATP production, significant increase in ROS generation, and diminished antioxidant defense. Mitochondrial biogenesis declines with age due to alterations in mitochondrial dynamics and inhibition of mitophagy, an autophagy process that removes dysfunctional mitochondria. Age-dependent abnormalities in mitochondrial quality control further weaken and impair mitochondrial function. In aged tissues, enhanced mitochondria-mediated apoptosis contributes to an increase in the percentage of apoptotic cells. However, implementation of strategies such as caloric restriction and regular physical training may delay mitochondrial aging and attenuate the age-related phenotype in humans.

## 1. Introduction

Mitochondrial dysfunction, including decreased oxidative capacity and increased oxidative damage, is thought to substantially contribute to biological aging. A fundamental impact of mitochondria on aging has been suggested several decades ago. One concept considers aging as the result of an accumulation of damage to biomolecules due to the excessive production of highly toxic reactive oxygen species (ROS). This concept was developed as the mitochondrial theory of aging since mitochondria are the major producers of ROS in the cell [1]. According to this theory, with age, mitochondria accumulate ROS-induced damage and become dysfunctional. With time, the function of cells declines causing aging and subsequent death. This concept was supported by a growing body of experimental data from animal models. For example, mice developed to have high mutation rates in mitochondrial DNA (mtDNA) (so called mtDNA mutator mice) exhibited advanced aging phenotypes [2]. On the other hand, many recent studies have also provided data contradicting this theory. For example, the knockout of superoxide dismutase genes did not affect the lifespan of* Caenorhabditis elegans* [3]. Indeed, the role of mitochondria in aging seems to be very complex.

Mitochondria are subcellular self-autonomous organelles primarily responsible for the generation of energy and ATP synthesis. Besides this, mitochondria play an essential role in amino acid and lipid metabolism and regulation of apoptosis. Mitochondria have their own DNA; however, it encodes only 1% of the approximately 1,000 mitochondrial proteins. A vast majority of mitochondrial proteins are encoded by nuclear DNA and are transported to mitochondria from the cytoplasm. Mitochondria may change in their numbers and mass due to the dynamic processes such as fission and mitophagy. Mitophagy is a specific form of autophagy that is required to degrade dysfunctional or damaged mitochondria [4].

In this review, we briefly consider the major changes in the function and dynamics of mitochondria that make them dysfunctional and contribute to aging.

## 2. Changes in Mitochondrial DNA in Aging

The mitochondrial theory of aging is based on the fact that mitochondrial DNA (mtDNA) has a higher rate of mutation and less efficient repair machinery compared to nuclear DNA. The mutation rate of mtDNA is up to 15-fold higher than that of nuclear DNA [5]. Indeed, the accumulation of mutations in mtDNA may reach a critical threshold and cause adverse effects especially in mitochondria in which improperly functioning or damaged components of the respiratory chain need to be replaced. Mutations in mtDNA that alter the expression of oxidative phosphorylation (OxPhos) complexes can lead to mitochondrial dysfunction and accelerated ROS generation [6]. Development of the mtDNA mutator mouse, an animal with mutated mtDNA polymerase *γ*, highlighted the strong potential for mtDNA mutations in aging. These mice had a defective mechanism in their mtDNA proofreading during replication and resulted in the generation of a large number of new mutations and the development of premature aging phenotypes [2]. According to the “vicious cycle” concept, mtDNA mutations are accumulated exponentially and should be associated with marked burst in ROS production [7]. However, experiments involving mtDNA mutator mice have shown a linear progression in the accumulation of mtDNA mutations over the lifespan. There were no significant changes in ROS production and activity of antioxidant enzymes in the mtDNA mutator mice compared to the normal animals [8]. Indeed, these findings seriously compromise the “vicious cycle” theory that suggests that mtDNA mutations and impaired OxPhos but not ROS production are primarily responsible for premature aging in the mtDNA mutator mice.

In addition, due to the close proximity to the ROS-producing components of the respiratory chain and the absence of histones, mtDNA is highly prone to oxidative damage. In rat hepatocytes, the amount of 8-hydroxydeoxyguanosine, a marker of DNA oxidative damage, was 16 times higher in mtDNA than in the nuclear DNA [9]. In the skeletal muscles and liver of rats, substantial age-related reductions in mtDNA copy number were observed [10]. These findings suggest that the amount and integrity of mtDNA may decline with age and lead to aberrant expression of electron transport chain proteins, thereby impairing the mechanism of OxPhos [5].

## 3. Mitochondrial ROS Production and Aging

The electron transport chain located in the inner mitochondrial membrane consists of four protein complexes and is coupled with ATP synthase, an ATP-producing enzyme. ROS are considered to be unwanted and toxic by-products of the mitochondrial electron transport system.

Due to their extreme reactivity, ROS seem to be a major mediator of age-associated cellular damage. ROS can also act as signaling molecules [11]. Interestingly, low doses of ROS could actually promote longevity while high doses, in contrast, shorten the lifespan of* C. elegans* [12]. A paradoxical increase in longevity was observed in mitochondrial respiration mutants of* C. elegans* at elevated levels of ROS. ROS were shown to activate hypoxia-induced factor-1 (HIF-1), a transcription factor associated with prolonged lifespan [12]. Mild inhibition of mitochondrial respiration was shown to extend lifespan in many species such as* C. elegans*,* Drosophila,* and mice, suggesting that an increase in longevity by moderate suppression of mitochondrial respiration is evolutionarily preserved.

Antioxidant enzymes involved in ROS inactivation provide protection against oxidative stress. Indeed, defects in the activity of mitochondrial antioxidant enzymes may increase oxidative stress. Mice containing a transgene of a mitochondrial antioxidant enzyme such as Mn-dependent superoxide dismutase (Mn-SOD) or catalase showed increased longevity [13, 14] while mice lacking Mn-SOD died from premature death associated with severe mitochondrial dysfunction and neurodegeneration [15]. Mice deficient in p66shc, a protein involved in mitochondrial ROS production independent from OxPhos mechanism, displayed advanced resistance to oxidative stress and an increase in lifespan by 30% [16].

Enzymatic changes may affect mitochondrial oxidative capacity and ATP synthesis. In humans, ATP-producing capacity decreases by 8% per decade [5]. Similarly, elderly people were found to have a 1.5-fold reduction in oxidative capacity per mitochondrial volume and a 1.5-fold reduction per muscle volume [17]. Age-dependent decline in mitochondrial function may result from low physical activity because when physical activity is compared between old and young people, most studies failed to find any significant correlations between age, mitochondrial respiration, and ATP flux [18, 19].

## 4. Age-Dependent Changes in Mitochondrial Dynamics 

The mitochondrial dynamics include the movement of mitochondria along the cytoskeleton, the regulation of mitochondrial architecture, and connectivity mediated by fusion/fission events [20]. This dynamic network is essential to maintain normal mitochondrial functions and participates in fundamental processes including aging. Mitochondrial biogenesis is the expansion of mitochondria through mechanisms involving growth (increase in mitochondrial mass) and division (increase of mitochondrial number).

With advanced age, the mitochondrial density in skeletal muscle was shown to decline gradually [21] and may suggest a decrease in mitochondrial biogenesis. The decline in mitochondrial biogenesis may result from an age-dependent reduction in levels of PGC-1*α*, a key regulator of biogenesis [22]. In aged mice, overexpression of PGC-1**α** in skeletal muscles was associated with reduced sarcopenia and an improvement of mitochondrial function [23].

Impaired balance between fission and fusion events may also be related to age-dependent decline in mitochondrial biogenesis. Fission is important for maintaining mitochondrial quality and integrity since it is involved in the selection of dysfunctional mitochondria. Defective mitochondria fail to function properly and have an impaired oxidative capacity skewed toward increased ROS production. These mitochondria are selectively removed by mitophagy, an autophagy-lysosome system that degrades dysfunctional mitochondria through fusion with lysosomes [4]. With age, mitophagy was observed to decline [24]. This decline is associated with an accumulation of damaged mitochondria, advanced oxidative stress, and increased apoptosis [25].

## 5. Mitochondrial Apoptotic Pathway and Aging

Mitochondria-mediated apoptosis is induced in response to proapoptotic stimuli or in the case of severe failure in OxPhos. Briefly, a caspase-dependent mechanism of mitochondrial apoptosis is accompanied by the release of cytochrome c and other factors from mitochondria, which then triggers the activation of a cascade of irreversible apoptotic events mediated by caspases. In the caspase-independent pathway, the release of endonuclease G and apoptosis-inducing factor (AIF) by mitochondria leads to the degradation of nuclear DNA [26].

Apoptosis was shown to increase significantly with age as reflected by an age-dependent gain in the percentage of apoptotic cells [27] and significant upregulation of caspase-independent proapoptotic pathway in aged rats [28] and elderly people [29]. Since no significant changes in the expression of caspases in older subjects were observed [29, 30], the caspase-dependent mechanism is unlikely to be activated with advanced age.

## 6. Genetic and Structural Alterations of Mitochondria in Atherogenesis

Increasing age is well known as an independent risk factor for the development of atherosclerosis [31, 32], and, therefore, according to a well-established point of view, atherosclerosis can be considered as a disease of aging [31, 32]. Premature or accelerated vascular aging and atherosclerosis can be associated with dysfunction of mitochondria [33, 34].

**Figure 1:**
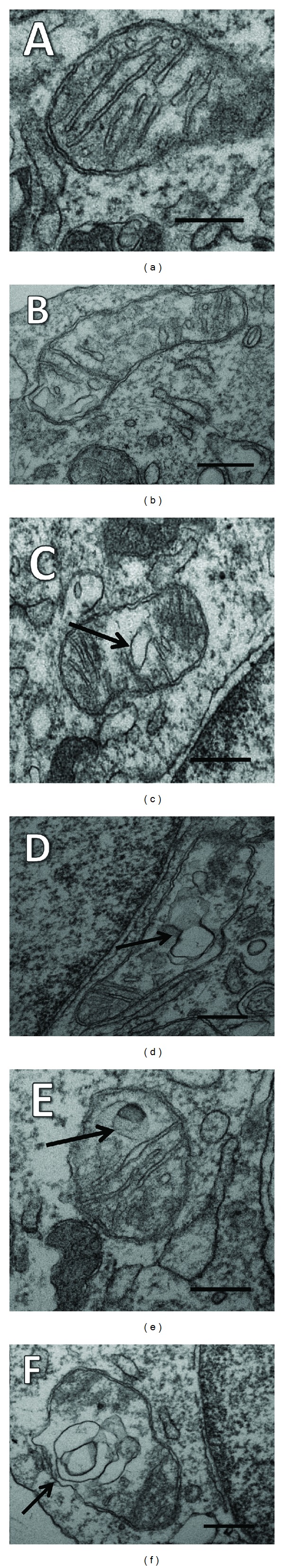
Different ultrastructural appearances of mitochondria in the aortic intima ((a)–(f)). (a) A typical appearance of a mitochondrion in a grossly normal aorta. (b) A mitochondrion with well-defined cristae and well-preserved surrounding membranes in a lipofibrous plaque. ((c)–(f)) Structural variants and destructive alterations of cristae of mitochondria in lipofibrous plaques. In ((c)–(f)), the formation of vacuole-like structures in zones of oedematous matrix of mitochondria is shown by arrows. ((a)–(f)) Electron microscopy scales = 200 nm (reprinted from Atherosclerosis; Sobenin et al. Changes of mitochondria in atherosclerosis: possible determinant in the pathogenesis of the disease 2013; 227 : 283–288 [38], with permission from Elsevier).

It is well known that in human pathology, a number of diseases are associated with somatic mutations in the mitochondrial genome (mtDNA) [35, 36]. Even though mitochondrial dysfunction leads to increased oxidative stress, the role of mitochondrial mutations in atherosclerosis has not received much attention so far [33, 34]. In a recent study we analyzed the association of mitochondrial genetic variation with the severity of carotid atherosclerosis (as assessed by carotid intima-media thickness (cIMT) and the presence of coronary heart disease (CHD)) and found that heteroplasmy for several mutations in the mtDNA in leukocytes, including C3256T, T3336C, G12315A, G13513A, G14459A, G14846A, and G15059A mutations, were significantly associated with both the severity of carotid atherosclerosis and the presence of CHD [37]. Electron-microscopic analysis of atherosclerotic lesions has also revealed a high variability in the ultrastructural appearance of mitochondria in human aortic atherosclerotic lesions compared with the appearance of mitochondria in the normal parts of the aortic intima (Figure 1) [38]. This prompted us to hypothesize that the structural variations in the appearance of mitochondria might reflect the existence of somatic mutations in the human mitochondrial genome which could be a determinant of the development of atherosclerotic lesions [38]. To test this hypothesis, we have compared the levels of heteroplasmy for several mitochondrial mutations previously proposed to be associated with different types of atherosclerotic lesions [38]. The homogenates of unaffected aortic intimae and lipofibrous plaques of human aortas were compared to reveal the average level of heteroplasmy for A1555G, C3256T, T3336T, G12315A, G14459A, and G15059A mutations of human mitochondrial genome [38]. It has been found that at least four mutations of mitochondrial genome, namely, A1555G in MT-RNR1 gene, C3256T in MT-TL1 gene, G12315A in MT-TL2 gene, and G15059A in MT-CYB gene, have significantly higher prevalence and mean value in lipofibrous plaques as compared to nonatherosclerotic intima [38]. The findings that somatic mutations in the mitochondrial genome are associated with the development of atherosclerosis [37, 38] should encourage further exploration of the concept that mitochondrial DNA heteroplasmy might be used as a biomarker of atherogenesis.

## 7. Mitochondrial Quality Control and Aging

Mitochondria have a number of specific enzymes such as chaperones, proteases, and methionine reductase to refold and eliminate misfolded proteins [39]. In the short-lived fungus* Podospora anserina*, deletion of mitochondrial protease PaLon1 involved in protein quality control leads to significantly shortened lifespan while overexpression of PaLon1 did not affect fungus longevity but was associated with prolonged good health and improved mitochondrial function [40]. The mitochondrial protease Lon is responsible for degradation of oxidized proteins and its downregulation is suggested to contribute to aging and age-related diseases [41]. Thus, the proper function of the mitochondria-associated quality control system may be associated with longevity or at least extend the healthy lifespan.

Mitochondria contribute to the cellular system of the protein quality control associated with ubiquitination and protease-dependent degradation of unfolded proteins by degrading cytosolic proteins located at the outer mitochondrial membrane [42]. Generally, the ubiquitin-protease system activity was shown to decline with age in mammals [43]. However, expression of some components of this system such as ubiquitin-specific proteases and certain proteasome subunits is upregulated with aging while levels of other components remain unchanged or decrease [44]. In muscles of aged rats, the expression of proteasome-associated proteins was increased and levels of 26S proteasomes were found to be higher by 2- to 3-fold of those of adult animals. Indeed, age-dependent activation of the ubiquitin-protease system may contribute to enhanced degradation of myofibrillar proteins and age-related muscle atrophy. The specific role of mitochondrial-associated protein degradation in aging is not completely understood and needs to be characterized.

## 8. Conclusion

**Figure 2:**
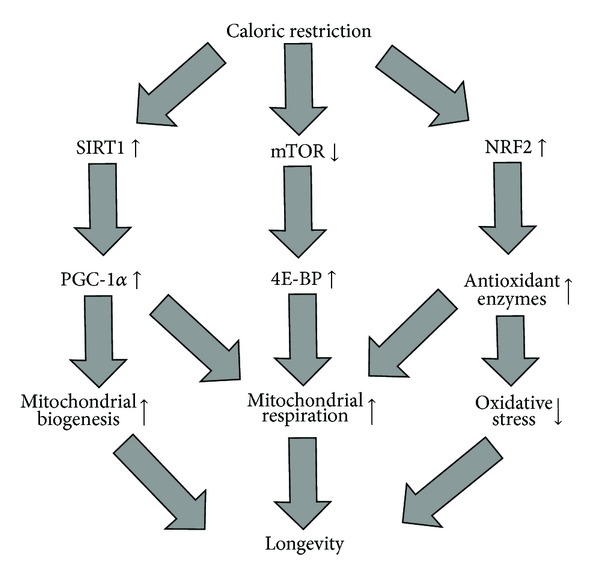
Mechanisms by which caloric restriction may improve mitochondrial function, delay mitochondrial aging, and expend longevity. Caloric restriction (CR) triggers several pathways that may lead to increased longevity via stimulation of mitochondrial function. The first mechanism includes the induction of sirtuin-1 (SIRT1), a protein deacetylase that in turn activates peroxisome proliferator-activated receptor-*γ* coactivator-*α* (PGC-1*α*). PGC-1*α* is a transcription factor involved in the activation of genes whose products are involved in mitochondrial biogenesis and respiration. CR also inhibits mammalian target of rapamycin (mTOR) signaling associated with an increase in the activity of eukaryotic translation initiation factor 4E binding protein (4E-BP) that stimulates the translation of genes encoding mitochondrial respiratory components. In* C. elegans*, CR activates the nuclear factor-erythroid 2-related factor-2 (NRF2) that regulates expression of several antioxidant genes and therefore may lengthen* C. elegans* lifespan through the reduction of oxidative stress and improving mitochondrial respiration.

It is clear that a role of mitochondria in aging is more complicated than suggested by the mitochondrial theory of aging. Multiple changes in mitochondrial function, structure, distribution, and dynamics contribute to aging or age-related features. Studies in model organisms such as yeast,* C. elegans*,* Drosophila*, and mice have shown that both suppression and stimulation of mitochondrial function can extend lifespan. For example, downregulation of mTOR signaling associated with enhanced protein synthesis and cell growth was shown to increase longevity in model organisms through improved mitochondrial efficiency and better energy consumption [45].  Figure 2 outlines the consequence of the mechanisms/factors by which caloric restriction may improve mitochondrial function, delay mitochondrial aging, and expand longevity.

As a consequence, caloric restriction (CR) that usually involves consuming 20–40% lower calories than normal was suggested as a promising intervention to increase both median and maximal lifespan in humans [46]. In a CR trial CALERIE based on 25% CR, CR patients were shown to have less mtDNA damage, more mtDNA content, and increased expression of some antioxidant enzymes, therefore suggesting that CR improves mitochondrial function and delays mitochondrial aging through reducing oxidative stress. The increase in expression of several proteins involved in mitochondrial biogenesis such as PGC-1*α*, Tfam, and SIRT1 was reported in CR patients compared to controls [47]. In summary, CR reduces oxidative stress and enhances mitochondrial biogenesis in order to produce mitochondria that are more efficient in ATP production, have optimal oxidative capacity, and generate less ROS.

Exercise training alone or in combination with CR may also represent an efficient strategy to delay mitochondrial aging and age-related dysfunction in humans through mechanisms stimulating mitochondrial biogenesis and oxidative capacity and improving protein quality control [48]. There is strong evidence that exercise training can optimize mitochondrial function in elderly individuals [49, 50]. Exercise, combined with a low carbohydrate (glycogen) diet, was shown to increase the expression of PGC-1*α* and optimize the oxidative capacity of human skeletal muscle [51]. In the CALERIE trial, combining CR with exercise training resulted in a 38% reduction in the estimated risk of cardiovascular disease, an important age-associated pathology, compared to controls [52]. Indeed, increased physical activity or even simply adopting active style habits may clearly reduce the rate of mitochondrial decline and attenuate the age-related phenotype.
